# A Novel Inflammation- and Nutrition-Based Prognostic System for Patients with Laryngeal Squamous Cell Carcinoma: Combination of Red Blood Cell Distribution Width and Body Mass Index (COR-BMI)

**DOI:** 10.1371/journal.pone.0163282

**Published:** 2016-09-22

**Authors:** Yan Fu, Yize Mao, Shiqi Chen, Ankui Yang, Quan Zhang

**Affiliations:** 1 Department of Head and Neck Oncology, Sun Yat-sen University Cancer Center, State Key Laboratory of Oncology in South China, Collaborative Innovation Center for Cancer Medicine, Guangzhou, China; 2 Department of Hepatobiliary Oncology, Sun Yat-sen University Cancer Center, State Key Laboratory of Oncology in South China, Collaborative Innovation Center for Cancer Medicine, Guangzhou, China; Shanghai Jiao Tong University School of Medicine, CHINA

## Abstract

**Background:**

Laryngeal squamous cell carcinoma (LSCC) is a head and neck cancer type. In this study, we introduced a novel inflammation- and nutrition-based prognostic system, referred to as COR-BMI (Combination of red blood cell distribution width and body mass index), for LSCC patients.

**Methods:**

A total of 807 LSCC patients (784 male and 23 female, 22–87 y of age) who underwent surgery were enrolled in this retrospective cohort study. The patients were stratified by COR-BMI into three groups: COR-BMI (0) (RDW ≤ 13.1 and BMI ≥ 25); COR-BMI (1) (RDW ≤ 13.1 and BMI < 18.5 or 18.5 ≤ BMI < 25; RDW > 13.1 and 18.5 ≤ BMI < 25 or BMI ≥ 25); or COR-BMI (2) (RDW > 13.1 and BMI < 18.5). Cox regression models were used to investigate the association between COR-BMI and cancer-specific survival (CSS) rate among LSCC patients.

**Results:**

The 5-y, 10-y, and 15-y CSS rates were 71.6%, 60.1%, and 55.4%, respectively. There were significant differences among the COR-BMI groups in age (< 60 versus ≥ 60 y; P = 0.005) and T stage (T1, T2, T3, or T4; P = 0.013). Based on the results, COR-BMI (1 versus 0: HR = 1.76; 95% CI = 0.98–3.15; 2 versus 0: HR = 2.91; 95% CI = 1.53–5.54, P = 0.001) was a significant independent predictor of CSS.

**Conclusion:**

COR-BMI is a novel inflammation- and nutrition-based prognostic system, which could predict long-term survival in LSCC patients who underwent surgery.

## Introduction

Laryngeal squamous cell carcinoma (LSCC) is a head and neck cancer type [1]. Approximately 20,875 new cases of LSCC were diagnosed, and 11,488 deaths from LSCC occurred in China in 2011[2]. In spite of recent advances in the field, the National Cancer Data Base reported that the survival rate of LSCC patients has decreased from 57.1% to 51.9% [1]. Therefore, it is important to assess the prognostic factors of patients with LSCC.

There are several clinical prognostic factors in LSCC patients. In clinical practice, the most commonly used predictor of survival in LSCC patients is the tumor-node-metastasis (TNM) classification system; however, its predictive ability is not ideal [3]. Several tumor biomarkers, such as EZH2, MMP11, and P14, may be used individually or combined in LSCC prognosis [4, 5]; however, these biomarkers are rarely used in routine clinical practice due to their high costs, lack of standardization, and limited availability. Therefore, there is a need to identify a valid and reliable clinical prognostic parameter.

Chronic inflammation, which is commonly present in malnourished patients, is associated with poor patient outcomes [6, 7]. The systemic inflammatory response in cancer patients contributes to reduced iron metabolism, diminished response to erythropoietin, and decreased red blood cell survival, which may explain the symptoms and clinical signs in cancer patients: weight loss, anorexia, and cancer-related anemia [8, 9]. Additionally, a poor nutritional status increases the risk of infection due to impaired immune function, delayed wound healing as a result of reduced collagen production, altered blood clotting rates, increased blood vessel wall fragility, and increased risk of postoperative complications [10–13].

Studies have reported that high red blood cell distribution width (RDW) and low body mass index (BMI) values are indicative of malnutrition and chronic inflammation in cancer patients [14, 15]. In this study, we first introduced a novel prognostic system, referred to as COR-BMI (Combination of Red Blood Cell Distribution Width and Body Mass Index), which assesses inflammation and nutritional status in LSCC patients. Inflammation and nutritional status are associated with LSCC prognosis [16, 17]; therefore, we hypothesized that preoperative COR-BMI scores predict LSCC patient outcome.

## Materials and Methods

### Study Population

**Fig 1:**
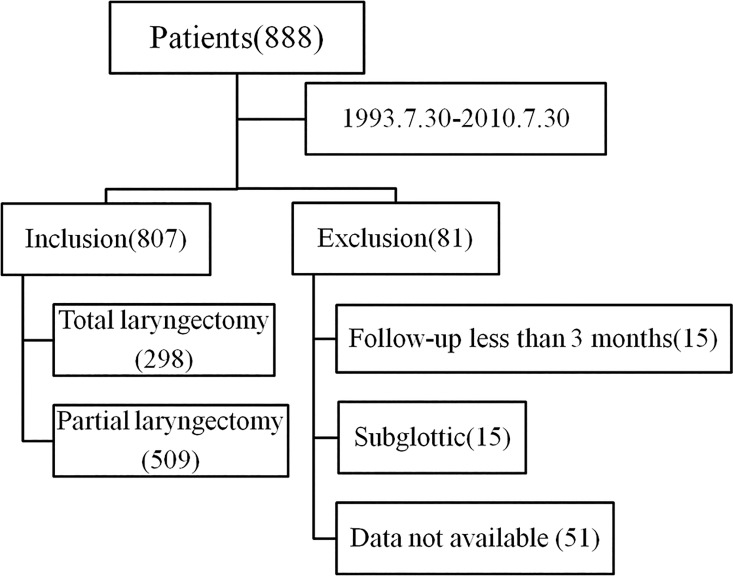
Patient selection process.

This study was approved by the Institutional Review Board of the Sun Yat-sen University Cancer Center (SYSUCC, Guangdong, China). This retrospective cohort study was conducted in Southern China. We retrospectively analyzed patients who underwent laryngectomy as a 1st curative treatment option for LSCC between July 30^th^, 1993 and July 30^th^ 2010 at SYSUCC. The exclusion criteria consisted of patients with incomplete preoperative laboratory data, patients with subglottic LSCC, and patients who were lost to follow-up < 3 months post-surgery. A total of 807 LSCC patients were included in the study (Fig 1).

### Data Collection

Clinical information was extracted from medical records at SYSUCC. Clinicopathological parameters included patient age (y), sex (male/female), smoking (no/yes), alcohol consumption (no/yes), neck dissection (no/yes), tumor subsite (glottic or supraglottic), T stage (T1–T4), N stage (N0–N3), histological differentiation (high/moderate/poor), height (m), and weight (kg). We used the conventional TNM staging system for cancer, which was established by the Union for International Cancer Control and the American Joint Committee on Cancer (AJCC) [18]. Laboratory data, including RDW, were obtained from preoperative examination. Cancer-specific survival (CSS) was defined as the time (in months) from the date of surgery until death due to LSCC. Follow-up evaluations were performed every three months during the first five years and annually thereafter.

### Determination of RDW, BMI, and COR-BMI Cut-Off Values

For the RDW, a cutoff of 13.1 was generated according to the receiver operating characteristic (ROC) analysis in the training set for CSS (sensitivity 61.9%, specificity 54.2%, area under the curve [AUC] 0.59, 95% CI 0.55–0.63, P < 0.001) (S1 Fig). RDW values were categorized into two groups: RDW ≤ 13.1 and RDW > 13.1. BMI was categorized into three groups: BMI < 18.5, 18.5 ≤ BMI < 25, and BMI ≥ 25[19]. Patients with RDW ≤ 13.1 and BMI ≥ 25 were defined as COR-BMI (0). Patients with RDW ≤ 13.1 and BMI < 18.5 or 18.5 ≤ BMI < 25, and patients with RDW > 13.1 and 18.5 ≤ BMI < 25 or BMI ≥ 25 were defined as COR-BMI (1). Patients with RDW > 13.1 and BMI < 18.5 were defined as COR-BMI (2).

### Statistical Analysis

RDW optimal cut-off values were determined by the ROC curve. Differences among the three COR-BMI groups (0, 1, and 2) were compared by χ^2^ test or Fisher’s exact test. Clinicopathological parameters that had significant effects (P < 0.05) on CSS based on univariate analyses were subjected to multivariate analyses using the Cox proportional hazards model. Hazard ratios (HRs) and the corresponding 95% confidence intervals (CIs) were estimated from Cox regression analysis. Patients’ clinical end points were calculated using the Kaplan-Meier method and compared by the log-rank test. For continuous variables, the data were expressed as mean ± SD. All analyses were carried out using IBM SPSS statistics software, version 20.0 (SPSS Inc., Chicago, IL, USA). P < 0.05 was considered significant.

## Results

A total of 807 LSCC patients enrolled in the study. The 5-y, 10-y, and 15-y CSS rates were 71.6%, 60.1%, and 55.4%, respectively. Among the 807 patients, 784 were male (97.1%) and 23 were female (2.9%). The median age was 60 y (22–87 y; Table 1). Approximately 90% of the patients had a history of smoking, and 34.3% of the patients consumed alcohol on a frequently basis. The most common tumor subsite was the glottic larynx (560, 69.4%), followed by the supraglottic larynx (247, 30.6%). The rate of cervical lymph node metastasis was 19.3%. Among all LSCC cases, 24.5% were in T1 stage, 30.6% were in T2 stage, 25.4% were in T3 stage, and 19.5% were in T4 stage. Among the 807 LSCC patients, 39.3%, 43.4%, and 17.3% had high, moderate, and poor differentiation, respectively.

**Table 1:** Patients’ Clinicopathological Characteristics.

Variables		COR-BMI 0(N %)	COR-BMI 1(N %)	COR-BMI 2(N %)	P value
**age**	**<60**	30 (50.0)	340 (50.9)	25 (31.6)	0.005
	**≥60**	30 (50.5)	328 (49.1)	54 (68.4)	
**Gender**	**Female**	1 (1.7)	19 (2.8)	3 (3.8)	0.756
	**Male**	59 (98.3)	649 (97.2)	76 (96.2)	
**Smoking status**	**No**	6 (10.0)	72 (10.8)	9 (11.4)	0.966
	**Yes**	54 (90.0)	596 (89.2)	70 (88.6)	
**Drinking status**	**No**	43 (71.7)	429 (64.2)	58 (73.4)	0.159
	**Yes**	17 (28.3)	239 (35.8)	21 (26.6)	
**Neck dissection**	**No**	40 (66.7)	481 (72.0)	56 (70.9)	0.675
	**Yes**	20 (33.3)	187 (28.0)	23 (29.1)	
**Tumor subsite**	**Supraglottic**	42 (70.0)	462 (69.2)	56 (70.9)	0.946
	**Glottic**	18(30.0)	206 (30.8)	23 (29.1)	
**T**	**1**	16 (26.7)	171 (25.6)	11 (13.9)	0.013
	**2**	23(38.3)	204 (30.5)	20 (25.3)	
	**3**	11 (18.3)	161 (24.1)	33 (41.8)	
	**4**	10 (16.7)	132 (19.8)	15 (19.0)	
**N**	**0**	50 (83.3)	539 (80.7)	62 (78.5)	0.855
	**1**	4 (6.7)	61 (9.1)	10 (12.7)	
	**2**	6 (10.0)	63 (9.4)	7 (8.9)	
	**3**	0 (0.0)	5 (0.7)	0 (0.0)	
**Histological Differentiation**	**high**	29 (48.3)	256 (38.3)	32 (40.5)	0.525
	**moderate**	20 (33.3)	295 (44.2)	35 (44.3)	
	**poor**	11 (18.3)	117 (17.5)	12 (15.2)	

Abbreviations: COR-BMI = Combination of red blood cell distribution width and body mass index; N = node; T = tumor

Based on COR-BMI scores, LSCC patients were further divided into three groups. Specifically, 60 (7.4%) of the patients had COR-BMI (0), 668 (82.8%) had COR-BMI (1), and 79 (9.8%) had COR-BMI (2). Based on χ2-test results, the only significant differences among the COR-BMI groups were in age (< 60 y versus ≥ 60 y; P = 0.005) and T stage (T1, T2, T3, or and T4; P = 0.013).

### Univariate and Multivariate Analyses of CSS

In univariate analysis, age (P = 0.001), alcohol consumption (P = 0.001), neck dissection (P < 0.001), tumor subsite (P < 0.001), T stage (P < 0.001), N stage (P < 0.001), histological differentiation (P < 0.001), and COR-BMI scores were significant predictors of CSS (Table 2). These factors were subsequently subjected to multivariate analyses using the Cox proportional hazards model. Based on the multivariate results, COR-BMI (1 versus 0: HR = 1.76; 95% CI = 0.98–3.15; 2 versus 0: HR = 2.91; 95% CI = 1.53–5.54, P = 0.001) was a significant independent predictor of CSS.

**Table 2:** Cox Regression Analyses for Cancer-specific Survival in Laryngeal Squamous Cell Carcinoma.

Characteristics	Univariate		Multivariate	
	HR (95%CI)	P Value	HR(95% CI)	P value
**Age**				
<**60**	1 (reference)	0.001	1 (reference)	0.002
**≥60**	1.48 (1.18–1.86)		1.43 (1.13–1.80)	
**Gender**				
**Female**	1 (reference)	0.051	ND	ND
**Male**	0.38 (0.14–1.01)			
**Smoking**				
**No**	1 (reference)	0.139	ND	ND
**Yes**	1.35 (0.91–2.02)			
**Drinking**				
**No**	1 (reference)	0.001	1 (reference)	0.001
**Yes**	1.47 (1.17–1.84)		1.50 (1.19–1.89)	
**Neck dissection**				
**No**	1 (reference)	< 0.001		NS
**Yes**	2.01 (1.59–2.55)			
**Tumor subsite**				
**Supraglottic**	1 (reference)	< 0.001		NS
**Glottic**	1.85 (1.47–2.33)			
**T stage**				
**T1**	1 (reference)	< 0.001	1 (reference)	<0.001
**T2**	1.37 (0.96–1.94)		1.22 (0.85–1.73)	
**T3**	2.24 (1.59–3.15)		1.69 (1.18–2.41)	
**T4**	3.28 (2.31–4.66)		2.10 (1.43–3.09)	
**N stage**				
**N0**	1 (reference)	< 0.001	1 (reference)	<0.001
**N1**	2.69 (1.95–3.70)		1.81 (1.28–2.56)	
**N2**	2.51 (1.78–3.53)		1.81 (1.25–2.62)	
**N3**	18.68 (7.54–46.30)		11.71 (4.61–29.79)	
**Histological Differentiation**				
**high**	1 (reference)	< 0.001	1 (reference)	0.049
**moderate**	1.51 (1.17–1.95)		1.24 (0.95–1.61)	
**poor**	2.04 (1.50–2.79)		1.50 (1.08–2.08)	
**COR-BMI**				
**0**	1 (reference)	< 0.001	1 (reference)	0.001
**1**	1.92 (1.08–3.43)		1.76 (0.98–3.15)	
**2**	3.25 (1.72–6.16)		2.91 (1.53–5.54)	

Abbreviations: COR-BMI = Combination of red blood cell distribution width and body mass index; CI = confidence interval; HR = hazard ratio; N = node; T = tumor; ND = not done; NS = not significant.

### CSS Rates by COR-BMI Score

**Fig 2:**
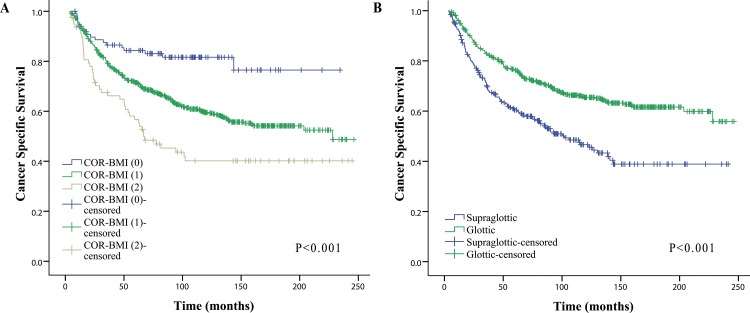
Kaplan-Meier curves for CSS rates of subgroups. (A) Kaplan-Meier curves for CSS rates of LSCC patients categorized by COR-BMI score (0/1/2). (B) Kaplan-Meier curves for CSS rates of LSCC patients categorized by tumor subsite. Abbreviations: CSS = cancer-specific survival; LSCC = Laryngeal squamous cell carcinoma; COR-BMI = Combination of red blood cell distribution width and body mass index.

The CSS rates of patients with COR-BMI (2) were significantly lower than those of patients with COR-BMI (1) and COR-BMI (0) (5-y CSS: 56.7% versus 72.5% and 81.0%, respectively; 10-y CSS: 40.2% versus 60.9% and 78.8%, respectively; 15-y CSS: 40.2% vs. 55.4% and 78.8%, respectively log-rank: P < 0.001; Fig 2A).

### Subgroup Analysis

The 5-y CSS, 10-y CSS and 15-y CSS rates for patients with supraglottic LSCC were 60.4%, 46.6% and 38.9%, respectively; these rates were lower than the 5-year, 10-y CSS and 15-y CSS rates of the glottic LSCC group (76.3%, 65.4% and 61.6%), the differences were statistically significant (P<0.001) (Fig 2B). The baseline characteristics of 807 patients with LSCC based on the stratification with the tumor subsite (see S1 Table). To further investigate prognostic factors in patients with LSCC, we conducted subgroup analysis for CSS in terms of tumor type (supraglottic LSCC group vs glottic LSCC group). Based on the multivariate results, COR-BMI was also a significant independent predictor of CSS for patients with supraglottic LSCC and glottic LSCC (supraglottic LSCC group:1 versus 0: HR = 3.62; 95% CI = 1.47–8.93; 2 versus 0: HR = 6.20; 95% CI = 2.25–17.07, P = 0.002; glottic LSCC group: 1 versus 0: HR = 1.67; 95% CI = 0.94–2.95; 2 versus 0: HR = 2.76; 95% CI = 1.41–5.39,P = 0.008) (see S2 Table).

### Neutrophil-to-Lymphocyte Ratio (NLR), Hemoglobin (Hb), T Stage, and COR-BMI

**Fig 3:**
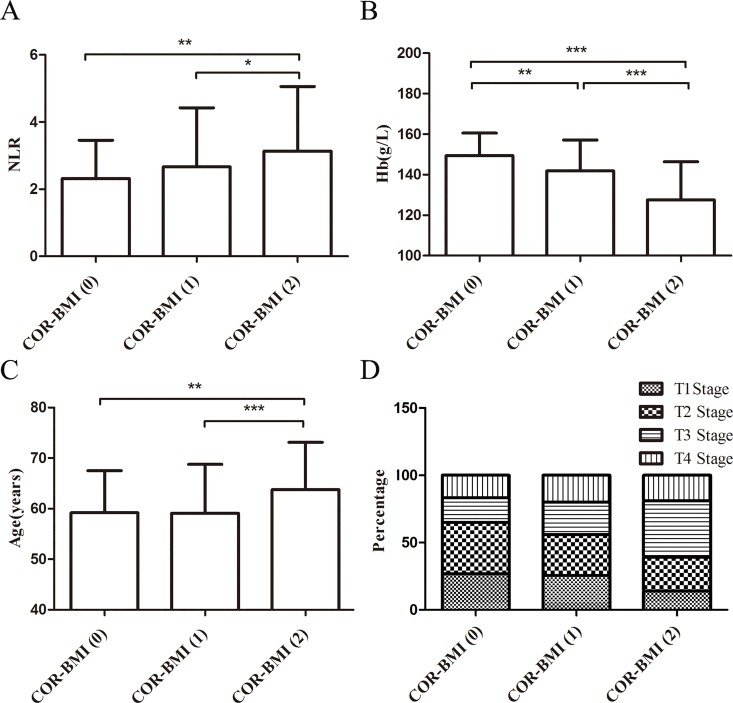
The values or distribution of parameters were various in different COR-BMI scores. (A) -(C) The data were expressed as mean ± SD. (A) NLR in patients with different COR-BMI scores. (B) Hb levels in patients with different COR-BMI scores. (C) Age of patients with different COR-BMI scores. (D) T stage of patients with different COR-BMI scores. (*:0.01<p<0.05;**:0.001<p<0.01;***:p<0.001.) Abbreviations: COR-BMI = Combination of red blood cell distribution width and body mass index; NLR = neutrophil-to-lymphocyte ratio; Hb = hemoglobin; T = tumor stage.

NLR and Hb levels increased with increasing COR-BMI scores (Fig 3A and 3B). As shown in Fig 3C, the average age of patients with COR-BMI (2) was higher than that of patients with COR-BMI (0) or COR-BMI (1). With increasing COR-BMI scores, the number of advanced T stage (T3 and T4) significantly increased (Fig 3D).

## Discussion

This study evaluated the relationship between COR-BMI scores and CSS rates in LSCC patients who underwent surgery. There were three COR-BMI scores: COR-BMI (0), characterized by a high probability of survival; COR-BMI (1), characterized by a medium probability of survival, and COR-BMI (2), characterized by a low probability of survival. To the best of our knowledge, this is the first study that evaluated the association between preoperative COR-BMI scores and CSS rates in LSCC patients.

Chronic inflammation has been identified as a risk factor in several types of cancers [20–23]. Cancer-associated inflammation plays a significant role in the proliferation, angiogenesis, invasion, and migration of cancer cells [24, 25]. Studies have shown that high RDW and low BMI values are indicative of chronic inflammation in cancer patients [14, 15]. Inflammation affects RDW values by impairing iron metabolism, inhibiting the response to erythropoietin, and decreasing red blood cell survival via the production of inflammatory markers [9].Like RDW, low BMI is also a risk factor in cancer patients with inflammation [15]. Accumulating evidence indicates that component of the systemic inflammatory response, for example NLR, has been associated with prognosis of various cancers [26, 27]. NLR is a biomarker of inflammation; high NLR values are correlated with poor clinical outcomes in patients [28]. In this study, NLR values increased with increasing COR-BMI scores, which suggested that there may be a relationship between COR-BMI scores and chronic inflammation. Nutritional status, which is associated with chronic inflammation, is linked to the long-term outcomes of several types of cancers [15, 29]. The metabolic effects of cancer-induced inflammation result in weight loss [30]. Overweight and obese patients have higher nutritional reserves to get through cancer therapy; however, several head and neck cancer patients experience significant weight loss at the time of diagnosis [29, 31, 32]. Different biomarkers of malnutrition have been used to predict outcome in cancer patients, such as RDW and BMI. RDW may be indicative of nutritional deficiencies, especially of iron, vitamin B12, and folic acid, which are common in cancer [33, 34]. In addition, low BMI is a prognostic factor of long-term survival in patients with malignant tumors, including head and neck cancers. Specifically, low BMI is associated with low CSS rates [35]. Recent studies have shown that low BMI is linked with reduced insulin levels, due to low or impaired secretion of insulin-like growth factor I and sex steroids, which suppress cellular proliferation and stimulate apoptosis [36, 37]. Furthermore, it has been reported that LSCC patients with low baseline Hb levels have low CSS rates [38, 39]. Interestingly, in this study, Hb levels decreased with COR-BMI scores. Therefore, COR-BMI is associated with nutritional status.

To evaluate the usefulness of COR-BMI in the prognosis of LSCC, we used ROC curve for survival prediction to verify the optimal cut-off points for RDW. CSS rates among LSCC patients can be divided into two groups based on RDW values: RDW ≤ 13.1 and > 13.1. Increased RDW is associated with poor prognosis in cancer patients [14]. According to WHO, there are three weight categories: underweight (BMI < 18.5), normal weight (18.5 ≤ BMI < 25) and overweight (BMI ≥ 25)[19]. Low BMI values are associated with increased risk of cancer mortality in patients [40]. Based on univariate and multivariate analyses, COR-BMI was a significant independent predictor of CSS in LSCC patients. Moreover, by univariate and multivariate analyses, COR-BMI was also a significant independent predictor of CSS of the patients with supraglottic LSCC and glottic LSCC separately.

Recent studies have shown that age at first diagnosis is the most significant factor that affects prognosis in LSCC patients [41]. The older the patient, the lower the nutritional status. Our results confirmed that the average age of patients with COR-BMI (2) was higher than that of patients with COR-BMI (0) or COR-BMI (1).

Ramroth et al. [41] showed that tumor stage is a significant risk factor in LSCC. Specifically, T3- and T4-stage patients have a two-fold risk of dying compared to T1- and T2-stage patients. With a rapid disease progression, nutritional deficiencies emerge early contributing to a poor disease prognosis[30]. In our study, the proportion of T3 and T4 patients (60.85%) with COR-BMI (2) was higher than that of patients with COR-BMI (0) (35.0%) or COR-BMI (1) (43.9%).

These findings suggest that high COR-BMI scores are predictive of an aggressive LSCC phenotype that contributes to low CSS rates. RDW and BMI are biomarkers of inflammation that are easy to measure and could be performed before surgery.

Our study had some limitations. First, our study had a retrospective design that included 807 patients from a single institution. Therefore, our study findings need to be validated using a larger cohort of patients. Second, several studies have used different RDW cut-off values, which need to be verified [42–45]. Third, COR-BMI, as a novel inflammation- and nutrition-based prognostic system, may be assessed in conjunction with other inflammatory and nutritional biomarkers in LSCC patients, including NLR, C-reactive protein, and Hb. This requires further research.

## Conclusion

In conclusion, this is the first study that investigated the prognostic role of COR-BMI in LSCC patients. COR-BMI is an inflammation- and nutrition-based prognostic system of LSCC patients that is easy to measure.

## Supporting Information

S1 Fig ROC analysis based on RDW for cancer-specific survival. In this model, sensitivity was 61.9% and specificity was 54.2%. The AUC was 0.59 (95% CI0.55–0.63, P < 0.001).
(TIF)

S1 Table The baseline characteristics of 807 patients with LSCC based on the stratification with the tumor subsite. (DOCX)

S2 Table Cox Regression Analyses for Cancer-specific Survival in LSCC patients based on the stratification with the tumor subsite. (DOCX)
