# Supplementary figures and images for: A Novel Inflammation- and Nutrition-Based Prognostic System for Patients with Laryngeal Squamous Cell Carcinoma: Combination of Red Blood Cell Distribution Width and Body Mass Index (COR-BMI)
Source: PLoS One. 2016 Sep 22;11(9):e0163282. doi: 10.1371/journal.pone.0163282 (PMC5033418; doi:10.1371/journal.pone.0163282)

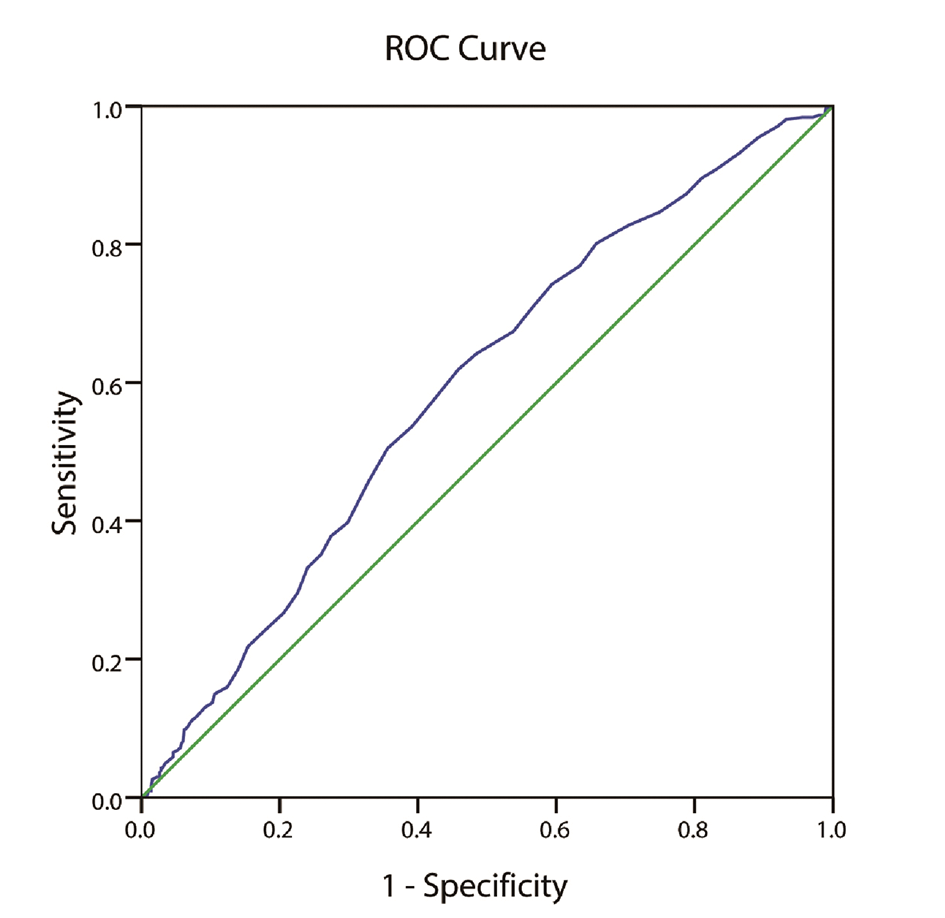

Supplement: S1 Fig — In this model, sensitivity was 61.9% and specificity was 54.2%. The AUC was 0.59 (95% CI0.55–0.63, P < 0.001). (TIF) [file pone.0163282.s001.tif]
