# Supplementary material for: A Novel Inflammation- and Nutrition-Based Prognostic System for Patients with Laryngeal Squamous Cell Carcinoma: Combination of Red Blood Cell Distribution Width and Body Mass Index (COR-BMI)
Source: PLoS One. 2016 Sep 22;11(9):e0163282. doi: 10.1371/journal.pone.0163282 (PMC5033418; doi:10.1371/journal.pone.0163282)
Supplement: S1 Table — (DOCX) [file pone.0163282.s002.docx]

| Variables | | Supraglottic LSCC (n=247) | | | | Glottic LSCC (n=560) | | | |
| --- | --- | --- | --- | --- | --- | --- | --- | --- | --- |
|  |  | COR-BMI 0  (N %) | COR-BMI 1  (N %) | COR-BMI 2  (N %) | P value | COR-BMI 0  (N %) | COR-BMI 1  (N %) | COR-BMI 2  (N %) | P value |
| age | **<60** | 17 (58.6) | 99 (50.8) | 8 (34.8) | 0.219 | 35 (50) | 219 (50.5) | 17 (30.4) | 0.017 |
|  | **≥60** | 12 (41.4) | 96 (49.2) | 15 (65.2) |  | 35 (50) | 215 (49.5) | 39 (69.6) |  |
| Gender | **Female** | 0 (0) | 9 (4.6) | 2 (8.7) | 0.311 | 2 (2.9) | 9 (2.1) | 1 (1.8) | 0.898 |
|  | **Male** | 29 (100) | 186 (95.4) | 21 (91.3) |  | 68 (97.1) | 425 (97.9) | 55 (98.2) |  |
| Smoking status | **No** | 4 (13.8) | 20 (10.3) | 2 (8.7) | 0.808 | 13 (18.6) | 41 (9.4) | 7 (12.5) | 0.069 |
|  | **Yes** | 25 (86.2) | 175 (89.7) | 21 (91.3) |  | 57 (81.4) | 393 (90.6) | 49 (87.5) |  |
| Drinking status | **No** | 16 (55.2) | 115 (56.1) | 13 (56.5) | 0.913 | 48 (68.6) | 293 (67.5) | 45 (80.4) | 0.148 |
|  | **Yes** | 13 (44.8) | 80 (41) | 10 (43.5) |  | 22 (31.4) | 141 (32.5) | 11 (19.6) |  |
| Neck  dissection | **No** | 7 (24.1) | 73 (37.4) | 8 (34.8) | 0.376 | 57 (81.4) | 384 (88.5) | 48 (85.7) | 0.240 |
|  | **Yes** | 22 (75.9) | 122 (62.6) | 15 (65.2) |  | 13 (18.6) | 50 (11.5) | 8 (14.3) |  |
| T | **1** | 2 (6.9) | 10 (5.1) | 0 (0) | 0.199 | 27 (38.6) | 148 (34.1) | 11 (19.6) | 0.061 |
|  | **2** | 10 (34.5) | 42 (21.5) | 3 (13) |  | 25 (35.7) | 150 (34.6) | 17 (30.4) |  |
|  | **3** | 8 (27.6) | 75 (38.5) | 14 (60.9) |  | 10 (14.3) | 79 (18.2) | 19 (33.9) |  |
|  | **4** | 9 (31) | 68 (34.9) | 6 (26.1) |  | 8 (11.4) | 57 (13.1) | 9 (16.1) |  |
| N | **0** | 15 (51.7) | 104 (53.3) | 9 (39.1) | 0.742 | 65 (92.9) | 405 (93.3) | 53 (94.6) | 0.382 |
|  | **1** | 5 (6.3) | 42 (21.5) | 7 (30.4) |  | 1 (1.4) | 17 (3.9) | 3 (5.4) |  |
|  | **2** | 9 (31) | 46 (23.6) | 7 (30.4) |  | 4 (5.7) | 10 (2.3) | 0 (0) |  |
|  | **3** | 0 (0.0) | 3 (1.5) | 0 (0.0) |  | 0 (0) | 2 (0.5) | 0 (0) |  |
| Histological type | **1** | 7 (24.1) | 35 (17.9) | 7 (30.4) | 0.614 | 41 (58.6) | 202 (46.5) | 25 (44.6) | 0.389 |
|  | **2** | 14 (48.3) | 99 (50.8) | 11 (47.8) |  | 21 (30) | 181 (41.7) | 24 (42.9) |  |
|  | **3** | 8 (27.6) | 61 (31.3) | 5 (21.7) |  | 8 (11.4) | 51 (11.8) | 7 (12.5) |  |

Table S1. The baseline characteristics of 807 patients with LSCC based on the stratification with the tumor subsite
