# Supplementary material for: A Novel Inflammation- and Nutrition-Based Prognostic System for Patients with Laryngeal Squamous Cell Carcinoma: Combination of Red Blood Cell Distribution Width and Body Mass Index (COR-BMI)
Source: PLoS One. 2016 Sep 22;11(9):e0163282. doi: 10.1371/journal.pone.0163282 (PMC5033418; doi:10.1371/journal.pone.0163282)
Supplement: S2 Table — (DOCX) [file pone.0163282.s003.docx]

| Characteristics | Supraglottic LSCC (n=247) | | | Glottic LSCC (n=560) | | |
| --- | --- | --- | --- | --- | --- | --- |
|  | Univariate | Multivariate | | Univariate | Multivariate | |
|  | P Value | HR (95%CI) | P Value | P Value | HR(95% CI) | P value |
| Age | | | | | | |
| ＜60 | 0.027 |  | NS | 0.005 | 1 (reference) | 0.008 |
| ≥60 |  |  |  |  | 1.49 (1.11-2.01) |  |
| Gender | | | | | | |
| Female | 0.121 | ND | ND | 0.079 | ND | ND |
| Male |  |  |  |  |  |  |
| Smoking | | | | | | |
| No | 0.189 | ND | ND | 0.384 | ND | ND |
| Yes |  |  |  |  |  |  |
| Drinking | | | | | | |
| No | 0.003 | 1 (reference) | 0.001 | 0.229 | ND | ND |
| Yes |  | 1.94 (1.33-2.84) |  |  |  |  |
| Neck dissection | | | | | | |
| No | 0.069 | ND | ND | 0.001 |  | NS |
| Yes |  |  |  |  |  |  |
| T stage | | | | | | |
| T1 | 0.001 | 1 (reference) | 0.018 | <0.001 |  | NS |
| T2 |  | 4.99 (0.67-37.30) |  |  |  |  |
| T3 |  | 5.80 (0.79-42.55) |  |  |  |  |
| T4 |  | 9.01 (1.22-66.30) |  |  |  |  |
| N stage | | | | | | |
| N0 | <0.001 | 1 (reference) | 0.003 | <0.001 | 1 (reference) | <0.001 |
| N1 |  | 1.71 (1.09-2.68) |  |  | 2.88 (1.65-5.01) |  |
| N2 |  | 1.69 (1.07-2.65) |  |  | 2.72 (1.31-5.66) |  |
| N3 |  | 6.48 (1.93-21.78) |  |  | 97.58(19.56-486.67) |  |
| Histological type | | | | | | |
| 1 | 0.885 | ND | ND | <0.001 | 1 (reference) | 0.002 |
| 2 |  |  |  |  | 1.39 (1.01-1.92) |  |
| 3 |  |  |  |  | 2.12 (1.39-3.23) |  |
| COR-BMI | | | | | | |
| 0 | 0.002 | 1 (reference) | 0.002 | 0.003 | 1 (reference) | 0.008 |
| 1 |  | 3.62 (1.47-8.93) |  |  | 1.67 (0.94-2.95) |  |
| 2 |  | 6.20 (2.25-17.07) |  |  | 2.76 (1.41-5.39) |  |

Table S2. Cox Regression Analyses for Cancer-specific Survival in LSCC patients based on the stratification with the tumor subsite
